# On Diseases of the Lungs and Pleuræ, Including Consumption

**Published:** 1887-04

**Authors:** 


On Diseases of the Lungs and Pleuræ, Including Consumption. By R. Douglas Powell, M. D., London, Fellow of the Royal College of Physicians; Physician to the Middlesex Hospital and Joint-Lecturer on Practical Medicine at the Medical School; Physician to the Hospital for Consumption and Diseases of the Chest at Brompton; late Assistant Physician and Lecturer on Materia Medica at Charing Cross Hospital. Third edition. Re-written and enlarged with illustrations. New York: William Wood & Co., 56 and 58 LaFayette Place. 1886.

The author's apology for devoting so much attention to treatment serves to recommend this book to the practical reader, and, indeed, the use of curative measures is the great end and aim of investigating the etiology and pathology of diseases.

The elegant colored plates encountered immediately after the table of contents illustrate a very important phase of the connection of tubercle-bacilli with phthisis pulmonalis, and reflect credit upon the artist.

Many of the ordinary complications found in the course of thoracic troubles are described minutely, so as to guide the practitioner in diagnosis and in the application of remedies, whether climatic or medicinal, and the wide reach of the work must render it a most acceptable contribution to our literature upon this class of diseases.

